# Congenital CMV Infection: Determination of Transplacental Passage of Aciclovir by Ex Vivo Placental Perfusion

**DOI:** 10.1111/1471-0528.70168

**Published:** 2026-02-01

**Authors:** Helyett Ollivier, Valentine Faure Bardon, Leo Froelicher Bournaud, Isidore Gaubert, Tiffany Guilleminot, Marianne Leruez‐Ville, Jean‐Marc Treluyer, Yves Ville, Gabrielle Lui, Julien Stirnemann

**Affiliations:** ^1^ Fetal Medicine and Obstetric Department, APHP Necker‐Enfants Malades Hospital Paris France; ^2^ Department of Obstetrics and Gynecology, Groupe Hospitalier Pitié Salpêtrière Université Pierre et Marie Curie‐Paris 6 Paris France; ^3^ Pharmacology and Therapeutic Evaluation in Children and Pregnant Women Paris Cité University, Inserm Paris France; ^4^ Perinatal, Pediatric, and Adult Pharmacology Department, Cochin Hospital Georges Pompidou European Hospital, Assistance Publique Hôpitaux de Paris Paris France; ^5^ CNRS, Institut Galien Paris‐Saclay, Université Paris‐Saclay Orsay France; ^6^ URP7328, Federation for Research Into Innovative Explorations an Therapeutics in Utero Paris, URP 7328 University of Paris Cité Paris France; ^7^ Virology Laboratory Necker‐Enfants Malades Hospital, Assistance Publique‐Hôpitaux de Paris Paris France; ^8^ Clinical Research and Pharmacology, AP‐HP, APHP‐CUP Cochin Necker Hospitals Paris France

**Keywords:** aciclovir, congenital CMV, fetal therapy, placental perfusion, placental transfer, valaciclovir

## Abstract

**Objective:**

To quantify the transplacental transfer of aciclovir at an amount equivalent to 2 g of valaciclovir corresponding to the fractionated dosing regimen given four times daily used to reduce congenital cytomegalovirus (CMV) transmission and to treat the CMV‐infected fetus.

**Design:**

Experimental ex vivo study.

**Setting:**

Dual closed‐loop perfusion of isolated human placental cotyledons.

**Population:**

Placentas collected at term.

**Methods:**

Placental transfer was assessed using the dual closed‐loop ex vivo perfusion. Aciclovir was perfused in the maternal compartment at a concentration corresponding to the oral dose of 2 g of valaciclovir taken every 6 h (8 g/day). Samples from maternal and fetal compartments were collected at regular intervals over a 3‐h period. Aciclovir concentrations were measured using chromatographic techniques. The transfer rate was calculated as the ratio of the number of moles of fetal aciclovir to the number of fetal and maternal moles at 3 h.

**Results:**

Nine perfusion experiments met the criteria for success and could be used for interpretation. The mean transplacental transfer rate of aciclovir was 17.4% (SD: 7.8%). With a single 2 g dose, fetal exposure to aciclovir remained below the IC50 for CMV.

**Conclusion:**

The transplacental transfer of aciclovir is low. Despite aciclovir's low molecular weight and hydrophilicity, moderate plasma protein binding and rapid renal elimination may limit placental availability, and the observed transfer was lower than expected for passive diffusion, suggesting involvement of facilitated uptake or efflux mechanisms. These results support the current rationale for high‐dose valaciclovir regimens in pregnancy and suggest a potential role for transporter‐mediated drug transfer.

## 1 Introduction

Congenital cytomegalovirus infection (CMV) affects 0.5%–2.0% of pregnancies annually [1]. Following maternal infection, the risk of transplacental transmission in early pregnancy is estimated at 30%–40% [2]. Fetal infections occurring during the periconceptional period or the first trimester account for most disease burden. CMV is implicated in 10% of cerebral palsy cases and is the leading cause of non‐genetic sensorineural hearing loss [3], with around 30% of first‐trimester infections resulting in long‐term sequelae [1, 2].

Fetal therapy with valaciclovir [4] aims to reduce both the risk of fetal infection [5, 6] and viral load in confirmed cases. To reach the fetal brain, maternally administered antivirals must cross several anatomical and functional barriers, including the placental syncytiotrophoblast, the fetal blood–brain barrier, and subsequently the vascular, mesenchymal and neuronal tissue barriers within the brain [7, 8, 9].

Valaciclovir is a purine nucleoside analogue that is rapidly converted after oral administration to aciclovir [10], which selectively inhibits viral DNA polymerase in infected cells [11].

Although aciclovir has antiviral activity against all herpes viruses, its sensitivity varies markedly: much higher concentrations are required against CMV (IC50: 2.5–25.0 mg/L) than against HSV (IC50: 0.02 mg/L) [12, 13]. Consequently, high‐dose aciclovir has been explored in high‐risk settings. In renal transplant recipients, a meta‐analysis by Sturgill MG et al. reported a favourable efficacy and safety profile compared with foscarnet and ganciclovir [14]. Similarly, in allogeneic haematopoietic stem cell transplantation, Kabut et al. observed a benefit restricted to CMV‐seropositive donor–recipient pairs (R+/D+), with reactivation rates reduced to 24% compared with previously reported rates of 30%–50% [15]. Pregnancy‐specific experimental models further inform dose–exposure relationships: using first‐trimester trophoblast cultures and third‐trimester ex vivo placental explants, Hamilton et al. showed that aciclovir displays antiviral activity in placental tissue only at relatively high concentrations compared with CMV‐specific antivirals [16].

While maternal pharmacokinetics of valaciclovir and aciclovir are well described [17], fetal transfer rate has been little invastigated ex vivo. Population pharmacokinetic studies have measured drug concentrations in maternal plasma, placenta, amniotic fluid and fetal blood [17, 18]. In an in vivo clinical study by Jacquemard et al. [18] the mother‐to‐fetus concentration ratio was 6.58 (i.e., a transfer rate of 15%), while the ratio of the average mother‐to‐fetus concentration was 1.42 (the mean maternal and fetal concentrations being 24.63 µmol and 17.38 µmol respectively), indicating an accumulation of aciclovir in the fetal compartment particulary in the amniotic fluid, with repeated doses.

Using an open‐loop perfused cotyledon model with lower aciclovir doses, Henderson et al. reported a 30% transfer of antipyrine [19]; however, closed‐loop systems more closely reflect physiological conditions and clinically relevant CMV dosing [20, 21].

Given the high doses required, potential maternal renal toxicity and compliance challenges, this study aimed to accurately determine the transplacental transfer rate of aciclovir using an ex vivo closed‐loop placental perfusion model [22].

## 2 Materials and Methods

### 2.1 Study Design

This was a prospective study conducted at the Necker Hospital maternity unit in Paris between 1 January 2024 and 1 November 2024. All women gave a signed consent. The study was approved by the ethics and research committee of the APHP (Assistance Public Hôpitaux de Paris): IRB N°IORG0010044.

We used placentas at > 37 weeks, whether the delivery occurred vaginally or by caesarean section. Exclusion criteria were the use of recent medication during delivery apart from the drugs commonly used for epidural analgesia and oxytocin, intrauterine growth restriction, preeclampsia, neonatal distress, chromosomal abnormalities, maternal general anaesthesia and twins.

For each placental perfusion, the following data were collected: date of birth, date of onset of pregnancy, gestational age, parity, existence or not of gestational diabetes, presence of maternal pathology, maternal smoking, date of delivery, route of delivery, type of locoregional anaesthesia, drugs given during delivery, possible carriage of type B streptococcus. Standard newborn biometric measurements, gender, pH and birth lactates were also recorded. Placenta were not selected based upon maternal CMV serostatus; however, all newborns underwent systematic salivary CMV screening, which was negative in all cases, excluding prenatal CMV transmission, including potential cervical shedding at delivery [23].

### 2.2 Placental Perfusion

**FIGURE 1:**
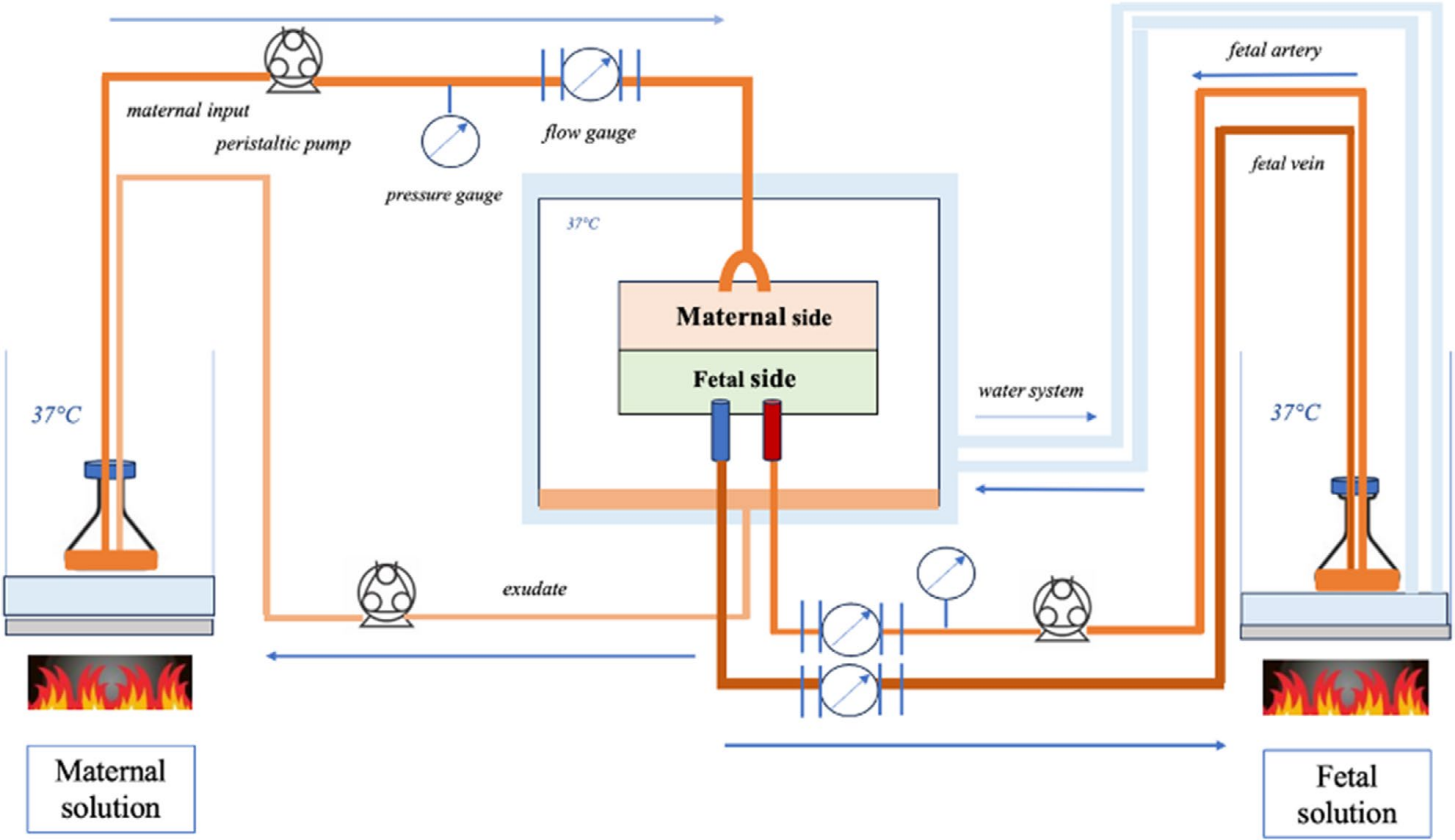
Schematic diagram of the closed‐loop for ex vivo placental perfusion.

All experiments were carried out within 30 min following delivery. Following careful macroscopic inspection of the placentas, a cotyledon was chosen and its vessels selectively perfused using the standardised Schneider closed circuit technique (Figure 1) [20, 21, 22]. The fetal artery and vein were catheterised using a 14 or 16 G needle catheter for each. The perfused cotyledon was then cannulated using two 16 G glass catheters fed by a pump connected to the maternal solution. The maternal arterial circuit was then established. The exudate formed during perfusion, representing the maternal vein, falls to the bottom of the chamber and is then pumped to the maternal solution to establish a closed circuit. The placental perfusion chamber was maintained at 37°C.

The maternal and fetal solutions consisted of EARLE's balanced salt solution (EBSS) at a concentration of 8.8 g/L (Earle's Balanced Salt 10 X, US Biological, Salem, MA, USA) diluted in 250 mL of sterile solution. After removing 37.5 mL of this solution, 37.5 mL of 20% albumin is added at the concentration of 30 g/L. Antipyrine (Merck, Germany), a small molecule of 118 Da which diffuses freely through the membrane, was used as a control marker for the experiment, and was added at the concentration of 20 mg/L to the maternal solution. The solution was buffered with sodium hydroxide to a target fetal pH of 7.2 and maternal pH of 7.4. The maternal and fetal solutions were placed in a water bath and a magnetic stirrer was used to mix the solutions continuously.

The perfused placentas were placed in the placental perfusion chamber, which was heated through a 37°C water circuit. The maternal and fetal solutions were maintained at 37°C in a water bath. The flow rate of the maternal solution imposed by a peristaltic pump was 12 mL/min and that of the fetus was 6 mL/min [24].

Each experiment was carried out over a period of 3 h. Fetal and maternal concentrations were monitored by taking 1 mL of maternal solution and 1 mL of fetal solution at predefined time points. 1 mL of maternal and 1 mL of fetal solution were sampled every 10 min for 30 min, then every 20 min for a period of 3 h for 2 placentas, and every 30 min for a period of 3 h for 7 placentas. To obtain additional data and to ensure that a steady state of transplacental transfer had been reached, the experiment was continued for a period of 5 h for one placenta, with samples taken every 30 min for up to 5 h. At each monitoring time point, the maternal and fetal volumes, the temperatures of the two solutions, the flow rates, pressure, and the pH values were recorded.

### 2.3 Aciclovir Dose

The medicines database of the French National Agency for the Safety of Medicines and Health Products (ANSM) provides pharmacokinetic data on aciclovir in the general population [25]: at a dose of 2 g of valaciclovir, the Cmax of aciclovir obtained is 8.3 mg/L (SD: 1.4 mg/L), as well as a population pharmacokinetic study in pregnant women infected with CMV reporting a mean Cmax of aciclovir at 9.2 mg/L (ranging between 6.1 and 19.8 mg/L) [17]. The dose of aciclovir provider prepared for each placenta is 2.1 mg, equivalent to a target concentration of 8.4 mg/L.

### 2.4 Dosage of Aciclovir and Antipyrine

Samples were stored at −20°C until analysis. Antipyrine concentrations were determined by high‐performance liquid chromatography with UV detection (HPLC‐UV) at 270 nm, on a Dionex UltiMate 3000 system (ThermoFisher Scientific, Les Ulis, France). In brief, 0.250 mL of each sample, standards (from 0.2 to 40.0 mg/L) and controls were mixed with paracetamol, the internal standard. Protein precipitation was achieved by adding acetonitrile, and the resulting supernatants were evaporated under nitrogen. The dried residues were then reconstituted in 0.100 mL of the mobile phase. Finally, 0.020 mL of each sample were injected into the HPLC‐UV system, using a C8 column (100 × 3 mm, 5 μm; CIL Cluzeau, Ste‐Foy‐la‐Grande, France). Chromatographic separation was performed using a mobile phase composed of 0.05 mol/L ammonium acetate buffer (pH adjusted at 5.07)—acetonitrile—methanol (75:13:12 [vol/vol/vol]). Analyte elution was achieved with a flow rate gradient ranging from 0.4 to 0.6 mL/min.

Aciclovir quantification was performed using a Dionex UltiMate 3000 system coupled to a TSQ Quantis triple quadrupole mass spectrometer (ThermoFisher Scientific, Les Ulis, France), equipped with a Heated Electrospray Ionisation (HESI‐II) probe in positive multiple reaction monitoring scan mode. In the reported procedures, [2H4]‐aciclovir was used as internal standard. Sample preparation was performed using a protein precipitation with methanol. After protein precipitation, all samples were centrifuged at 20000 *g* for 10 min, and 0.030 mL of the supernatant was diluted with 0.150 mL of water (0.05% formic acid, vol/vol). Finally, 0.020 mL of each sample was injected in the LC–MS/MS system. The separation was conducted on a Kinetex XB C18 analytical column (30 × 2.1 mm, 1.7 μm; Phenomenex, Le Pecq, France). The mobile phase consisted of water (0.05% of formic acid, vol/vol) and methanol (0.05% of formic acid, vol/vol). The analytes were eluted using a gradient for 8 min with a flow rate of 0.4 μg/mL. The following transitions for quantification were monitored: m/z 226.125 > 152.113 for aciclovir, m/z 230.162 > 152.125 for [2H4]‐aciclovir. The technique was validated according to European Medicines Agency (EMA) guidelines in the range of 0.05–20.00 mg/L.

### 2.5 Data Analysis

#### 2.5.1 Criteria for a Successful Placental Perfusion

The experiment was successful if placental perfusion lasted at least 3 h, if the fetal transfer rate of antipyrine (FTR) was at least 20%, and if the ratio at 3 h of fetal antipyrine concentration to maternal concentration was greater than 75%. Antipyrine is a small, not charged, lipophilic compound that crosses the placental barrier by passive diffusion; its transplacental transfer is flow‐dependent and is routinely used as an internal reference to verify the functional integrity of the perfusion system [24, 26, 27]. FTR and ratio were calculated using the following formulas:

$$\mathrm{FTR}=\frac{100\times \mathrm{FC}\times \mathrm{FV}}{\mathrm{FC}\times \mathrm{FV}+\mathrm{MC}\times \mathrm{MV}}$$

$$\text{Ratio}=\frac{\;\mathrm{FC}\;}{\mathrm{MC}},$$

where FC stands for Fetal Concentration, FV, Fetal Volume, MC, Maternal Concentration, MV: Maternal Volume.

The percentage aciclovir transfer rate was calculated using the following formula with concentration of aciclovir:

$$\mathrm{FTR}=\frac{100\times \mathrm{FC}\;180\times \mathrm{FV}180}{\mathrm{FC}180\times \mathrm{VF}180+\mathrm{MC}\;180\times \mathrm{MV}180}.$$

Raw data on the transplacental transfer rate for each placenta are available in the appendix.

Statistics were calculated using Excel software (version 16.79.2) and R software (version 4.4.0).

## 3 Results

Throughout the study, 9 placentas validated the criteria for transplacental transfer of antipyrine. The characteristics of the patients are presented in Table 1. The mean maternal age was 34 years old. Most women were multiparous (6/9, 67%), 2/9 (22%) were smokers, and deliveries were performed by caesarean section in 5/9 (56%) of women. None of the placentas were affected by peripartum acidosis (at the umbilical cord: mean pH: 7.28; lactate: 2.2 mmol/L).

**TABLE 1:** Characteristics of patients and newborns who underwent placental perfusion (*n* = 9).

Maternal age (years)	34.4 (SD: 5.2)
Nulliparous	3 (33%)
Smoking during pregnancy	2 (22%)
Spontaneous vaginal delivery	2 (22%)
Vaginal delivery after induction	2 (22%)
Emergency c‐section	4 (44%)
Elective c‐section	1 (12%)
Male newborn	7 (78%)
Birthweight (grams)	3259 (SD: 373)
Umbilical artery pH	7.28 (SD: 1.60)

The mean arterial and venous flows in the fetal circulation were at the target value of 6 mL/min, and the mean arterial flow in the maternal circulation was also at the target value of 12 mL/min. The average pH was at the target value of 7.2 (SD: 0.1) in the fetal solution and 7.4 (SD: 0.1) in the maternal solution. The average temperature of the fetal solution was 37.4°C (SD: 0.7°C), and that of the maternal solution was 37.3°C (SD: 0.5°C), for fetal and maternal targets at 37°C. In the human placenta, the average pressure in the fetal arteries is 50 mmHg [28]. In our experience, the average fetal pressure was 67.5 mmHg (SD: 30.9 mmHg); the absence of a significant increase in fetal blood pressure is indirect evidence of the absence or low incidence of thrombosis in the system. Finally, the average volume of the fetal solution at 3 h was 201 mL (SD: 57 mL) and that of the maternal solution was 247 mL (SD: 53 mL), ensuring minimal experimental leakage and fluid loss.

**FIGURE 2:**
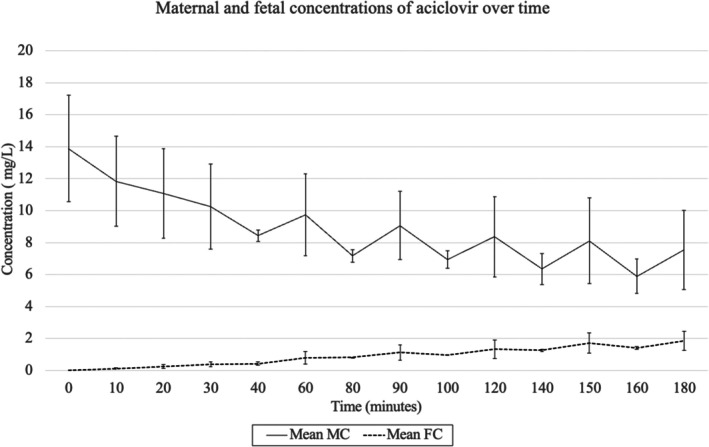
Maternal and fetal aciclovir concentrations over time. Mean FC, Mean Fetal concentration; Mean MC, Mean maternal Concentration.

The mean fetal transfer rate of antipyrine at 3 h was 81% (SD: 6.9%), and the maternal‐to‐fetal concentration ratio of antipyrine at 3 h was 39% (SD: 12.2%) (Table S1). The average maternal and fetal concentrations of aciclovir over time are shown in Figure 2. At 3 h, the average maternal concentration is 7.55 mg/L (SD: 2.46 mg/L), and the average fetal concentration is 1.85 mg/L (SD: 0.60 mg/L). Maternal concentrations of aciclovir decreased over time, as fetal concentrations increased.

Initial results from placentas perfused for 3‐h periods prompted us to carry out a 5‐h perfusion to ensure we had reached steady state. In the perfused placenta at 5 h, the concentration of aciclovir in the maternal compartment decreases over time between 3 and 5 h, varying from 12.42 mg/L at 3 h to 11.70 mg/L at 5 h.

The fetal concentration at 3 h was 2.86 mg/L, reaching a maximum of 3.47 mg/L at 270 min, then decreasing to 3.28 mg/L at 5 h.

The maternal and fetal concentrations in each placenta are shown in Figure S1 in the supporting information data.

**FIGURE 3:**
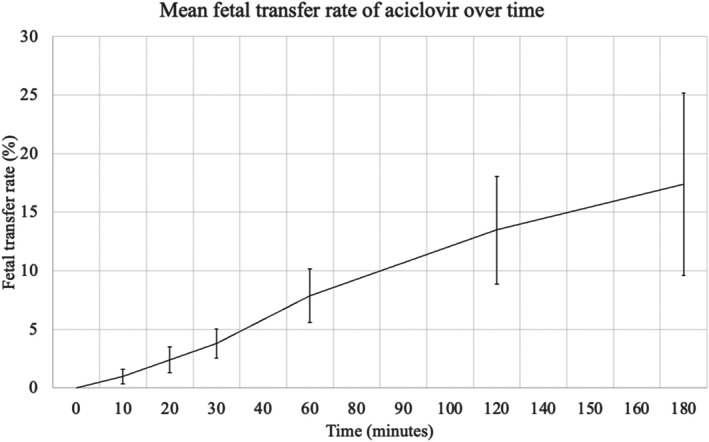
Mean fetal transfer rate of aciclovir over time.

The average fetal transfer rate of aciclovir between the maternal and fetal compartments was 17.4% at 3 h (SD: 7.8%). The results are presented in Figure 3.

## 4 Discussion

### 4.1 Mains Findings: Low Transplacental Transfer and Clinical Impact for Fetal Therapy

In this study, transplacental transfer was evaluated following a dose equivalent to a single 2 g dose of valaciclovir, corresponding to the fractionated dosing regimen currently recommended for CMV prophylaxis. This experimental design was chosen to reflect clinically relevant peak concentrations while accounting for the short half‐life of aciclovir (approximately 3 h), as a single 8 g bolus would not accurately reproduce clinical exposure conditions [18]. Using our ex vivo data, we observed a fetal transfer rate of approximately 17.4%. This finding suggests that, with a sigle 2 g dose, fetal exposure may remained about 26% below the therapeutic target. However, in the light of in vivo pharmacokinetic data from Jacquemard et al. obtained by cordocentesis during the second and third trimesters and showing that aciclovir accumulates in the fetal compartment particulary in the amniotic fluid, these results underscore the importance of a high‐dose regimen administered four times daily to promote a sufficient fetal exposure and achieve the IC50 in the fetal compartment.

Despite discordant in vitro findings suggesting pharmacokinetic limitations at the maternal‐fetal interface [17, 18], the clinical efficacity of the antiviral therapy in congenital CMV infection has been convincingly demonstrated in vivo. Clinical studies have shown a significant therapeutic benefit, indicating that these pharmacological constraints do not preclude meaningful antiviral activity in the fetus [5, 6].

Pregnancy‐specific experimental models further support the superior antiviral potency and favourable safety profile of CMV‐specific agents compared with aciclovir. Using complementary first‐trimester trophoblast cultures and third‐trimester ex vivo placental explants, Hamilton et al. showed that letermovir and maribavir have markedly lower EC50 values than aciclovir, without placental cytotoxicity at high concentrations [16]. Consistent with these data, the limited transplacental transfer observed in the present study suggests that alternative therapeutic strategies warrant consideration. Notably, letermovir and maribavir have been reported to exhibit low but measurable placental transfer (9% ± 1% and 10% ± 1%, respectively), while achieving fetal concentrations exceeding their IC50, as shown by Faure‐Bardon et al. [29].

### 4.2 Strengths and Limitations

The ex vivo dual perfusion model preserves the structural and functional integrity of the human placenta, providing a physiologically relevant system to study drug transfer without ethical concerns associated with in vivo studies [20, 28, 30, 31]. This approach allows assessment of placental drug kinetics and metabolism under controlled conditions. This model has been instrumental in evaluating the placental transfer of various pharmaceuticals, including antivirals, by simulating maternal and fetal circulations [31]. It allows for the assessment of drug kinetics, placental metabolism, and potential effects on placental tissue.

A degree of variability in fetal transfer rate was observed across the nine ex vivo perfusion experiments, with values at 3 h ranging from 7% to 31% (SD 7.8%). Such variability is inherent to human placental perfusion models and primarily reflects inter‐individual differences in placental structure and function and contributes to the physiological relevance and external validity of the model. Importantly, all placentas fulfilled predefined quality criteria, including appropriate antipyrine transfer, supporting the reliability of the experimental conditions.

Variations in initial maternal aciclovir concentrations at T0 were also observed and reflect practical constraints related to ex vivo experimental preparation. Notably, higher initial maternal concentrations were generally associated with higher fetal transfer, consistent with concentration‐dependent diffusion processes (Figure S2). However, these variations must be considered in the context of the intrinsic biological and experimental variability of human placental perfusion models.

Nevertheless, this technique makes it possible to reflect the transplacental transfer at the end of the 3rd trimester in pregnant women [31]. It would be difficult to extrapolate this raw result to the other trimesters of pregnancy, firstly because transfer by simple diffusion increases during pregnancy due to the disappearance of the cytotrophoblastic layer, and secondly because active and facilitated transfers depend on the presence and activity of transporters, which can vary according to gestational age [7, 9, 22]. Identifying the transporters of aciclovir across the transplacental membrane and their ontogenies during pregnancy could enable hypotheses to be made about transplacental transfer during the first and second trimesters of pregnancy.

We carried out a 5‐h experiment to ensure that we had achieved a state of equilibrium. Subject to the more limited interpretation of the data after 3 h of ex vivo perfusion (cellular apoptosis and acidosis), our results show that very little variation of the concentration takes place after 3 h, with the state of equilibrium appearing to be truly reached at around 260 min.

### 4.3 Interpretation

Data on the transplacental passage of aciclovir remain limited in the literature. In our ex vivo placental perfusion model, transfer was slightly lower than that reported by Henderson et al. [19], who estimated the transplacental passage of aciclovir to be about 30% of that of antipyrine (open‐loop experience). This discrepancy may reflect differences in experimental design, as open‐loop perfusion is less representative of in vivo physiology, and the aciclovir dose used by Henderson et al. was eightfold lower. They consequently proposed a mixed transfer mechanism involving both passive diffusion and transporter‐mediated processes.

Although the physicochemical properties of aciclovir—low molecular weight (225 Da) and hydrophilicity (Log P: −1.56)—would suggest efficient passive diffusion, other pharmacokinetic features may limit transfer, including moderate plasma protein binding (9%–33%) and rapid renal elimination, with predominant excretion unchanged [11]. Accordingly, the observed transfer rate of 17.4% suggests that passive diffusion alone does not fully explain aciclovir transplacental transfer.

As proposed by Henderson et al., aciclovir transfer may rely largely on carrier‐dependent mechanisms [19], and modelling of the present data could help clarify the relative contribution of active versus passive processes [32, 33]. In addition, placental efflux transporters may further restrict fetal exposure, potentially explaining the incomplete attainment of therapeutic concentrations in the fetal compartment with a single 2 g dose.

Aciclovir is a nucleoside analogue substrate of DNA polymerase that acts in competition with deoxyguanine triphosphate [34]. Although data on guanine transfer are scarce, animal studies suggest limited transplacental passage of purines and nucleosides [35]. Similar findings have been reported for entecavir, another nucleoside analogue with comparable physicochemical properties, which shows limited transplacental transfer in animal and in vitro models and appears to be a substrate for multiple transporters [36, 37, 38].

Future studies could build on this experimental system by evaluating aciclovir transplacental transfer in the presence of selective transporter inhibitors to assess the relative contribution of facilitated uptake and potential efflux mechanisms. In parallel, integration of these data into pharmacokinetic and mechanistic modelling frameworks would enable refinement of transplacental transfer parameters and estimation of the distinct rate constants governing the underlying transport processes.

## 5 Conclusion

Our findings are consistent with a fetal transfer rate of aciclovir at 3 h, at term,of 17.4%. These results suggest that transfer does not occur only by simple diffusion. The low fetal transfer rate combined with the very high IC50 of aciclovir for CMV justifies the high doses required to achieve therapeutic fetal concentrations.

## Author Contributions

Helyett Ollivier contributed to study conception, data collection, and manuscript drafting. Valentine Faure Bardon contributed to data analysis, provided methodological supervision, and critically revised the manuscript. Leo Froelicher Bournaud performed the aciclovir dosage and contributed to manuscript revision. Isidore Gaubert assisted with data analysis and revision of the manuscript. Tiffany Guilleminot was responsible for establishing the perfusion system, provided technical assistance, and contributed to manuscript revision. Jean‐Marc Treluyer, Marianne Leruez‐Ville, and Yves Ville critically revised the manuscript. Gabrielle Lui contributed to data analysis, performed the antipyrine dosage, provided technical assistance, and critically revised the manuscript. Julien Stirnemann contributed to study conception, data analysis, critical revision, and gave final approval of the manuscript.

## Funding

The authors have nothing to report.

## Ethics Statement

The study has been approved by the ethics and research committee of the APHP (Assistance Public Hôpitaux de Paris): IRB N°IORG0010044.

## Conflicts of Interest

The authors declare no conflicts of interest.

## Supporting information


**Figure S1:**. Maternal and fetal concentrations of aciclovir for each placenta as a function of time.

**Figure S2:** Fetal transfer rate for each placenta as a function of time.

**Table S1:** Fetal transfer rate of Antipyrine at 3 h for each placentas.

## Data Availability

The data that support the findings of this study are available on request from the corresponding author. The data are not publicly available due to privacy or ethical restrictions.
