# Supplementary figures and images for: Congenital CMV Infection: Determination of Transplacental Passage of Aciclovir by Ex Vivo Placental Perfusion
Source: BJOG. 2026 Feb 1;133(6):1241–8. doi: 10.1111/1471-0528.70168 (PMC13040426; doi:10.1111/1471-0528.70168)

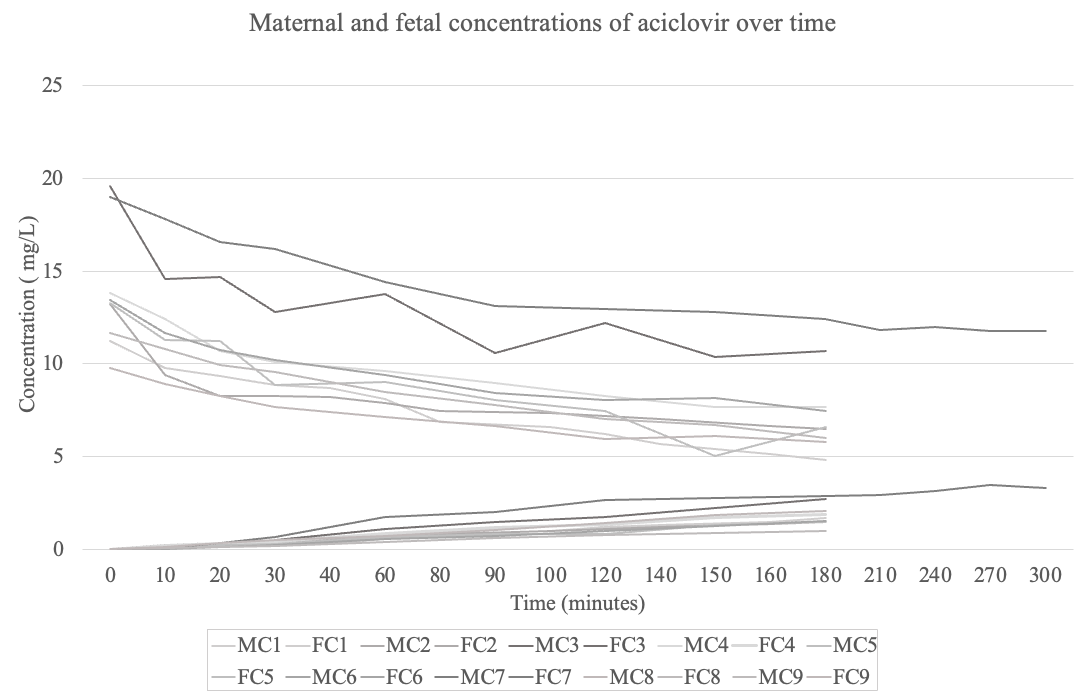

Supplement: Supplementary file 1 — Figure S1:. Maternal and fetal concentrations of aciclovir for each placenta as a function of time. [file BJO-133-1241-s002.png]

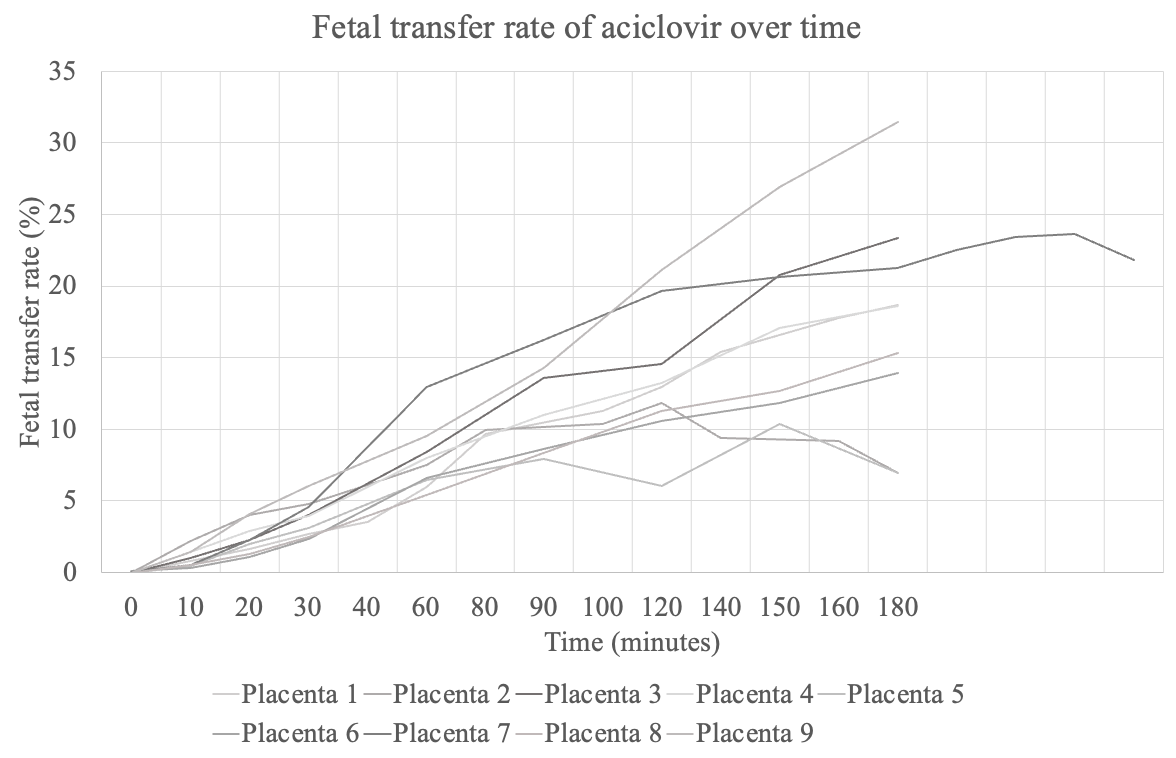

Supplement: Supplementary file 2 — Figure S2: Fetal transfer rate for each placenta as a function of time. [file BJO-133-1241-s001.png]
