# Supplementary material for: Congenital CMV Infection: Determination of Transplacental Passage of Aciclovir by Ex Vivo Placental Perfusion
Source: BJOG. 2026 Feb 1;133(6):1241–8. doi: 10.1111/1471-0528.70168 (PMC13040426; doi:10.1111/1471-0528.70168)
Supplement: Supplementary file 3 — Table S1: Fetal transfer rate of Antipyrine at 3 h for each placentas. [file BJO-133-1241-s003.docx]

**Supporting Information**

**Table S1: Fetal transfer rate of Antipyrine at 3 hours for each placentas**

|  | Maternal Concentration at 3 hours of Antipyrine  (µg/mL) | Fetal Concentration at 3 hours of Antipyrine (µg/mL) | Fetal Transfer Rate of Antipyrine (%) | Fetal concentration / Maternal concentration rate of Antipyrine (%) |
| --- | --- | --- | --- | --- |
| Placenta 1 | 8,09 | 7,07 | 36,81 | 87,39 |
| Placenta 2 | 10,11 | 7,96 | 20,79 | 78,73 |
| Placenta 3 | 8,69 | 6,77 | 48,32 | 77,91 |
| Placenta 4 | 7,79 | 6,45 | 43,70 | 82,80 |
| Placenta 5 | 12,09 | 9,25 | 20,08 | 76,51 |
| Placenta 6 | 11,23 | 8,72 | 37,80 | 77,65 |
| Placenta 7 | 14,38 | 10,73 | 46,72 | 74,62 |
| Placenta 8 | 8,45 | 6,51 | 44,52 | 77,04 |
| Placenta 9 | 6,85 | 6,59 | 56,19 | 96,20 |
| Mean | 9,74 | 7,78 | 39,44 | 80,98 |
| Standard Deviation | 2,43 | 1,50 | 12,19 | 6,87 |
